# Prediction of Prolonged Length of Hospital Stay After Cancer Surgery Using Machine Learning on Electronic Health Records: Retrospective Cross-sectional Study

**DOI:** 10.2196/23147

**Published:** 2021-02-22

**Authors:** Yong-Yeon Jo, JaiHong Han, Hyun Woo Park, Hyojung Jung, Jae Dong Lee, Jipmin Jung, Hyo Soung Cha, Dae Kyung Sohn, Yul Hwangbo

**Affiliations:** 1 Healthcare AI Team National Cancer Center Goyang Republic of Korea; 2 Department of Surgery National Cancer Center Goyang Republic of Korea; 3 Cancer Data Center National Cancer Control Institute National Cancer Center Goyang Republic of Korea; 4 Center for Colorectal Cancer, Research Institute and Hospital National Cancer Center Goyang Republic of Korea

**Keywords:** postoperative length of stay, cancer surgery, machine learning, electronic health records

## Abstract

**Background:**

Postoperative length of stay is a key indicator in the management of medical resources and an indirect predictor of the incidence of surgical complications and the degree of recovery of the patient after cancer surgery. Recently, machine learning has been used to predict complex medical outcomes, such as prolonged length of hospital stay, using extensive medical information.

**Objective:**

The objective of this study was to develop a prediction model for prolonged length of stay after cancer surgery using a machine learning approach.

**Methods:**

In our retrospective study, electronic health records (EHRs) from 42,751 patients who underwent primary surgery for 17 types of cancer between January 1, 2000, and December 31, 2017, were sourced from a single cancer center. The EHRs included numerous variables such as surgical factors, cancer factors, underlying diseases, functional laboratory assessments, general assessments, medications, and social factors. To predict prolonged length of stay after cancer surgery, we employed extreme gradient boosting classifier, multilayer perceptron, and logistic regression models. Prolonged postoperative length of stay for cancer was defined as bed-days of the group of patients who accounted for the top 50% of the distribution of bed-days by cancer type.

**Results:**

In the prediction of prolonged length of stay after cancer surgery, extreme gradient boosting classifier models demonstrated excellent performance for kidney and bladder cancer surgeries (area under the receiver operating characteristic curve [AUC] >0.85). A moderate performance (AUC 0.70-0.85) was observed for stomach, breast, colon, thyroid, prostate, cervix uteri, corpus uteri, and oral cancers. For stomach, breast, colon, thyroid, and lung cancers, with more than 4000 cases each, the extreme gradient boosting classifier model showed slightly better performance than the logistic regression model, although the logistic regression model also performed adequately. We identified risk variables for the prediction of prolonged postoperative length of stay for each type of cancer, and the importance of the variables differed depending on the cancer type. After we added operative time to the models trained on preoperative factors, the models generally outperformed the corresponding models using only preoperative variables.

**Conclusions:**

A machine learning approach using EHRs may improve the prediction of prolonged length of hospital stay after primary cancer surgery. This algorithm may help to provide a more effective allocation of medical resources in cancer surgery.

## Introduction

Cancer is a major burden on public health worldwide [1], and the amount of health care resources associated with its treatment is constantly increasing [2]. The major strategies of cancer treatment include surgery, chemotherapy, and radiation therapy, with surgery being the most common treatment approach. Compared with other cancer-related management strategies, cancer surgery requires greater use of health care resources and, consequently, greater medical costs [3]. Postoperative length of stay (POLOS) in the hospital is one of the reasons for the cost increase. As patients with cancer are discharged after full recovery from surgery, POLOS is also an indirect indicator of surgical recovery and postoperative complications in patients with cancer. That is, a prolonged POLOS (PPOLOS) indicates a delayed recovery after cancer surgery.

In previous literature, factors associated with PPOLOS have been evaluated for several cancer surgeries [4-8], and risk factors such as age, malnutrition, underlying diseases (including diabetes, cardiovascular diseases, renal dysfunction, and respiratory disease), and common blood count results (such as neutrophil-lymphocyte ratio, albumin, and hemoglobin) have been reported. However, the majority of studies have used a small number of subjects and have not evaluated a wide variety of clinical factors. Thus, there are many limitations to clinical application of the results of those studies.

Currently, most medical institutions store electronic health records (EHRs) and use them to improve the quality and efficiency of hospitals [9,10]. Many recent studies using EHRs have reported that machine learning–based models outperform statistical models in predicting outcomes and adverse events [11,12].

In this study, we assessed whether PPOLOS of patients with cancer can be predicted with machine learning approaches using EHR data and evaluated the effect of preoperative factors on the prediction of PPOLOS for each type of cancer.

## Methods

### Data Source and Subjects

Our retrospective study was conducted using EHR data from the Korea Cancer Big Data Platform (K-CBP), which was constructed in the National Cancer Center, Goyang, Republic of Korea. Details of the K-CBP have been described elsewhere [13]. Briefly, the K-CBP is a multidatabase framework that contains various medical information including clinical and genomic data and medical images. In this study, de-identified clinical data obtained from patients with cancer who visited the National Cancer Center were used. We extracted data from the K-CBP from 61,743 subjects with 19 cancer types who underwent primary cancer surgery between January 1, 2000, and December 31, 2017. The inclusion criteria for patients were as follows: (1) age ≥18 years, (2) surgery performed with general anesthesia, and (3) first instance of surgery for primary cancer. We excluded subjects who had emergency cancer surgery, cancer removal with local anesthesia, surgery for multiple primary cancers, or missing or typo-filled records for surgery, pathology, and hospitalization. Cancer types with fewer than 100 total cases were also excluded. Finally, we retrieved data from 42,751 subjects with 17 cancer types, including lip, oral cavity, and pharynx (oral; International Classification of Diseases codes C00-C14); esophagus (C15); stomach (C16); colon and rectum (colon; C18-C20); liver (C22); gallbladder and biliary tract (gallbladder; C23 and C24); pancreas (C25); larynx (C32); lung (C33 and C34); breast (C50); cervix uteri (C53); corpus uteri (C54); ovary (C56); prostate (C61); kidney (C64); bladder (C67); and thyroid (C73).

### Variables from EHRs

We examined several variables from diverse categories within EHRs, such as records of surgeries, blood tests, and medications, as well as pathologic reports and nursing charts. We only used medical data recorded within 6 months prior to surgery. For data on underlying diseases, only preoperative evaluation data were used. In the case of repeated data such as blood and biochemical tests, only the data recorded just before surgery was used in the analysis. For simplicity of interpretation, we reorganized variables into five major categories as follows: (1) surgical and cancer factors, (2) underlying diseases and functional laboratory assessments, (3) general assessments, (4) medications, and (5) social factors. Each major category consisted of one to five subcategories. Details of variables are described in Table 1. There were two types of missing values in our variables: missing numeric values were replaced by the middle value, and missing categorical values were replaced with “value unknown.” We conducted the min-max normalization for obtained whole variables. It should be noted that we basically used preoperative variables for the prediction of PPOLOS. However, tumor staging represented by T/N stage—based on the TNM staging system of the American Joint Committee on Cancer [14,15]—was extracted from pathologic reports because of the lack of structured T/N stage information in preoperative images. In addition, we obtained the operative time as a typical intraoperative factor and analyzed its effect on the prediction of PPOLOS.

**Table 1:** Preoperative clinical variables.

Major category and subcategory		Variables
**Surgical and cancer factors**		
	Surgery	Types of surgery Co-operations Surgeons
	Cancer stage	T/N stages
**Underlying diseases and functional laboratory assessments**		
	Underlying diseases and related laboratory parameters	Liver diseases (history of liver disease, hepatitis viral tests, aspartate aminotransferase, alanine aminotransferase, bilirubin, alkaline phosphatase, gamma-glutamyl transferase) Diabetes mellitus (history of diabetes, HbA_1c_^a^, glucose, urine glucose) Renal disease (history of renal disease, BUN^b^, creatinine) Cardiac disease (history of cardiac disease) Hypertension (history of hypertension) Allergic disease (history of allergic disease) Tuberculosis (history of tuberculosis) Cancer (history of cancer) Mental disorder (history of mental disorder)
	Cardiopulmonary functions	Pulmonary function (FVC^c^, FEV_1_^d^) Cardiac function (EF^e^, E/A^f^, RVSP^g^)
	Nutritional factors	Degree of appetite Albumin, globulin, A/G^h^ ratio, protein Cholesterol (total, LDL^i^, HDL^j^, triglyceride) Lymphocyte count
	Inflammatory factors	hs-CRP^k^, ESR^l^, fibrinogen
	Initial laboratory parameters	Blood count (except lymphocyte count) Electrolytes, chemistry tests Urinalysis Coagulation tests Hormone tests ABO blood type
**General assessments**		
	Demographic characteristics and anthropometric factors	Age Sex Height, weight, BMI Ambulation, ECOG^m^ performance Type of admission History of previous operation Family history of diseases Degree of diseases insight
	Vital signs	Blood pressure (systolic, diastolic) Body temperature Breath rate Pulse rate
	Substance exposure	Alcohol Smoking Alternative therapy
	Symptoms	Gastroenteric, cardiovascular, respiratory, neurologic, dermatologic, and urinary symptoms Sleep and fatigue Mood Pain
**Medications**		
	Drugs	Medications
**Social factors**		
	Family	Marriage, child, cohabitation
	Education	Level of education
	Religion	Type of religion
	Occupation	Type of job

^a^HbA_1c_: hemoglobin A_1c_.

^b^BUN: blood urea nitrogen.

^c^FVC: forced vital capacity.

^d^FEV_1_: forced expiratory volume in the first second of expiration.

^e^EF: ejection fraction.

^f^E/A: ratio of the early (E) to late (A) ventricular filling velocities.

^g^RVSP: right ventricular systolic pressure.

^h^A/G ratio: albumin to globulin ratio.

^i^LDL: low-density lipoprotein.

^j^HDL: high-density lipoprotein.

^k^hs-CRP: high-sensitivity C-reactive protein.

^l^ESR: erythrocyte sedimentation rate.

^m^ECOG: Eastern Cooperative Oncology Group.

### Definition of PPOLOS

In the literature, PPOLOS is defined in a variety of ways [7,8,16,17]. This study focused on predicting which patients with cancer will use a significant amount of hospital resources. Therefore, the PPOLOS study group was defined as the subset of patients who used 50% of the total ward after surgery. Specifically, we calculated the total number of postoperative bed-days by considering the respective length of stay between surgery and discharge for patients with each type of cancer. Next, we arranged the patients by POLOS from shortest to longest. Then, we defined the long-term hospitalized patient group, which occupied half of the total hospital bed-days, as the PPOLOS group.

### Models

To predict PPOLOS, we employed three models: (1) extreme gradient boosting (XGB) classifier [18], (2) multilayer perceptron (MLP) [19], and (3) logistic regression (LR). XGB classifier is one of the most widely used machine learning algorithms. It is a high-performance classifier based on gradient boosting that trains decision trees in succession such that residuals of earlier trees are corrected by later ones. MLP is a type of feed-forward neural network in which all computation is directed from the input layer to the output layer. The model is built on the architecture of at least three layers, with one input layer, variable hidden layers, and one output layer. Backpropagation is used to find optimal layer weights for the model [20]. LR is a commonly used classification algorithm to assign observations to a discrete set of classes. Unlike the majority of LR algorithms yielding continuous values, its outputs are converted by the sigmoid function into probabilities mapped to the classes. These models have been utilized in numerous medical and clinical studies to analyze EHRs, vital signals, and images, as well as to support medical decisions [21-24]. In our study, the MLP model consisted of a self-dot attention layer and two fully connected layers. We evaluated the performance of the model using 5-fold cross-validation. In each fold, training and test sets were divided in an 8:2 ratio.

## Results

### Ethics Statement

The research protocol was approved by the Institutional Review Board of the National Cancer Center (IRB No. NCC2018-0113). All data used in this retrospective study were de-identified.

### Characteristics of the Subjects

Multimedia Appendix 1 shows the characteristics of each cancer population. Stomach cancer surgery (n=8929) was the most common surgery in this study, followed by breast (n=8918), colon (n=7449), thyroid (n=5071), lung (n=4455), and liver (n=1342) cancer surgeries. The average age of the patients was 56.6 years, and women accounted for 55.75% (23,835/42,751) of the total cancer cases. Oral (mean 22.2 days, SD 22.3 days, median 16.9 days), esophageal (mean 22.1 days, SD 22.5 days, median 15.8 days), gallbladder (mean 20.7 days, SD 14.8 days, median 16.9 days), and pancreatic (mean 21.0 days, SD 15.1 days, median 16.9 days) cancers were associated with relatively long POLOS, whereas thyroid (mean 3.3 days, SD 2.2 days, median 3.0 days) and breast (mean 5.4 days, SD 6.5 days, median 4.1 days) cancers were associated with relatively short POLOS. The respective PPOLOS thresholds and proportions of patients with PPOLOS for each cancer type were as follows: stomach (10 days; 2481/8929, 27.80%), breast (6 days; 2354/8918, 26.40%), colon (11 days; 2143/7449, 28.77%), thyroid (4 days; 781/5071, 15.40%), lung (12 days; 1195/4455, 26.28%), liver (15 days; 320/1342, 25.34%), prostate (9 days; 312/1054, 29.60%), ovary (18 days; 266/1016, 26.18%), kidney (9 days; 162/767, 21.12%), esophageal (24 days; 184/761, 24.18%), cervix uteri (16 days; 150/706, 21.25%), corpus uteri (12 days; 120/535, 22.43%), oral (27 days; 113/528, 21.40%), gallbladder (25 days; 127/499, 25.45%), pancreatic (23 days; 99/365, 27.12%), bladder (11 days; 35/233, 15.02%), and larynx (31 days; 24/123, 19.51%).

### Prediction Performance

Multimedia Appendix 2 shows the performance of our models in predicting PPOLOS with four metrics: accuracy, specificity, sensitivity, and area under the receiver operating characteristic curve (AUC). When evaluating the AUC metrics for our XGB classifiers, the models performed excellently for kidney and bladder cancers (AUC >0.85). A moderate performance (AUC 0.70-0.85) was observed for stomach (AUC 0.83), breast (AUC 0.83), colon (AUC 0.71), thyroid (AUC 0.79), prostate (AUC 0.78), cervix uteri (AUC 0.78), corpus uteri (AUC 0.79), and oral (AUC 0.79) cancers. In contrast, the models had relatively low performance for lung, liver, ovary, esophageal, gallbladder, pancreatic, and larynx cancers (AUC <0.7).

**Figure 1:**
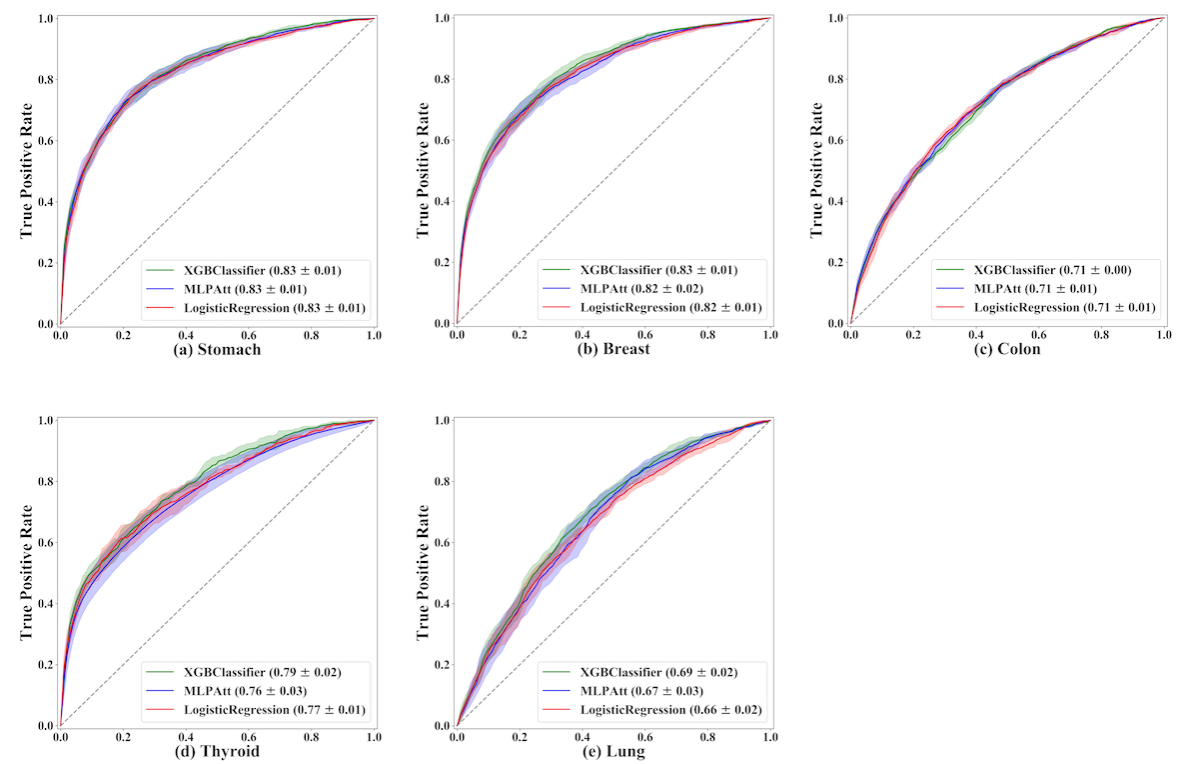
Receiver operating characteristic (ROC) curves of all models for major cancers. For each subfigure, a legend shows the average area under the ROCs with their standard deviations on 5-fold cross-validations for the models. Solid lines represent the plots of the average area under the ROCs, covering the plots of all area under the ROCs by its shaded regions. MLPAtt: multilayer perceptron with attention mechanism; XGB: extreme gradient boosting.

Receiver operating characteristic (ROC) curves of major cancers are shown in Figure 1. For cancers with fewer than 4000 cases, we found that classification performance did not vary significantly between the different models. However, for cancers with more than 4000 cases (stomach, breast, colon, thyroid, and lung cancers), the performance of XGB classifiers was superior to that of the other models. For the metric of sensitivity, which represents the prediction of cases with PPOLOS, MLP showed better performance than the other methods.

### Risk Factors of PPOLOS

We identified the most important variables for each model by examining the respective coefficient or attention score. Multimedia Appendix 3 shows the top 10 important variables from the models of the five cancers with the highest number of patients (stomach, breast, colon, thyroid, and lung cancers).

For each type of cancer, various risk factors were identified in the three models. The top 10 risk factors identified in the five cancers in the XGB classifier model were as follows:

- stomach cancer: albumin and globulin, urinary symptoms, surgeries (total gastrectomy and laparoscopy-assisted distal gastrectomy), forced expiratory volume in the first second of expiration, absolute neutrophil count, zolpidem use, and N stage;
- breast cancer: urinary symptoms, surgeries (modified radical mastectomy and breast-conserving surgery), surgeon, globulin, famotidine use, N stage, marriage, and metoclopramide use;
- colon cancer: surgeon, co-operation, albumin, surgeries (abdominoperineal resection and laparoscopic anterior resection), urinary symptoms, marriage, N stage, and urine white blood cell count;
- thyroid cancer: N stage, urinary symptoms, surgery (total thyroidectomy), albumin and globulin, ejection fraction, surgeon, drinking, and marriage; and
- lung cancer: albumin and globulin, sex, nonsmoker, absolute neutrophil count, theophylline use, route of admission, marriage, and hemoglobin.

No universal set of risk factors was present in subjects with PPOLOS, as the importance of a given variable was dependent on both the type of cancer and the model used.

### Contribution of the Variable Group to the PPOLOS Prediction

**Figure 2:**
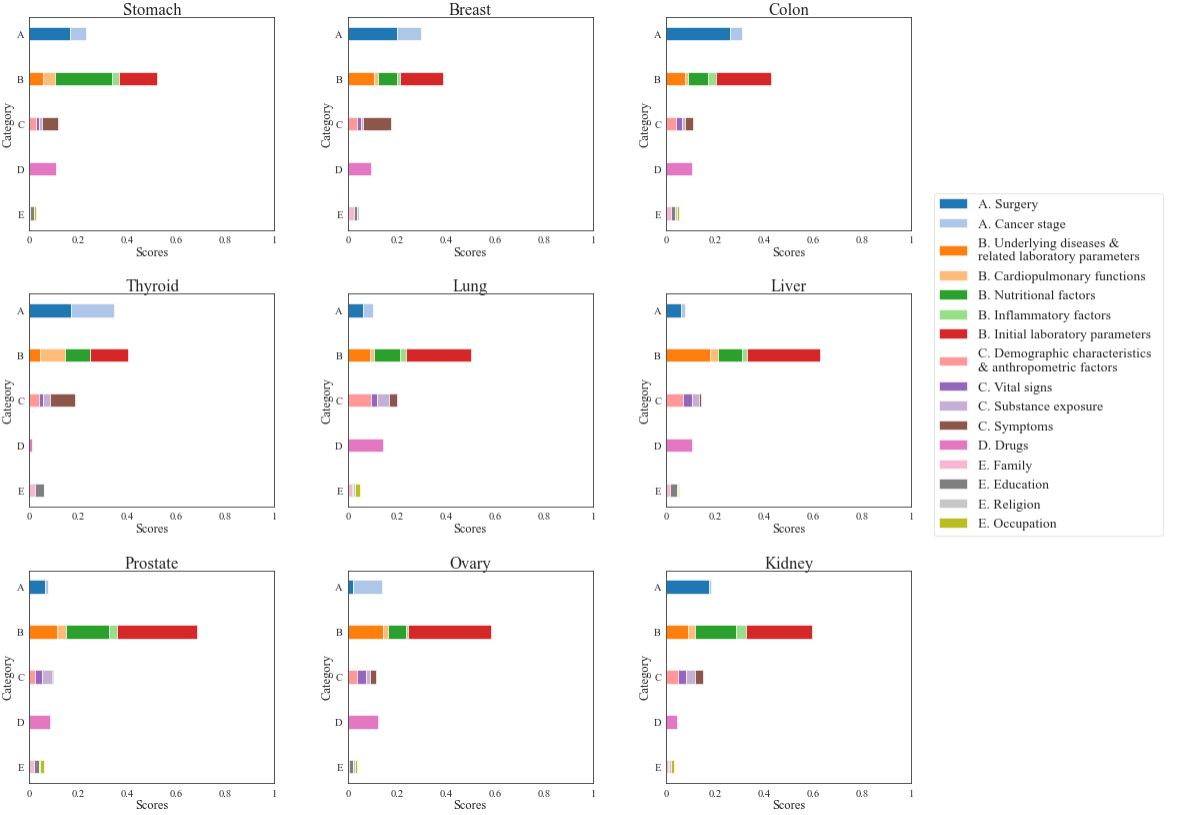
Variable scores derived from extreme gradient boosting (XGB) classifier for the top nine cancers of the patient population. Each subfigure shows cumulative scores of all variables derived from XGB classifier for a single cancer, where each bar is segmented into subcategories by colors and represents the cumulative score of a major category.

We plotted all variable scores derived from XGB classifier for nine types of cancer with the largest subject populations in Figure 2. In this figure, a bar represents the cumulative scores in a major category divided into colors corresponding to subcategories, with the sum of their cumulative scores equal to 1. We found that various variables contribute to the prediction of PPOLOS, which are different for each type of cancer.

We note that variables in major category A (ie, surgical and cancer factors) that occupy more than 20% of the total proportion influence the prediction of PPOLOS for the top four cancers of the patient population (ie, stomach, breast, colon, and thyroid) more than for the other five cancers. Major category B (ie, underlying diseases and functional laboratory assessments) contains the most influential factors for all nine cancers.

### Impact of the Operative Time

**Figure 3:**
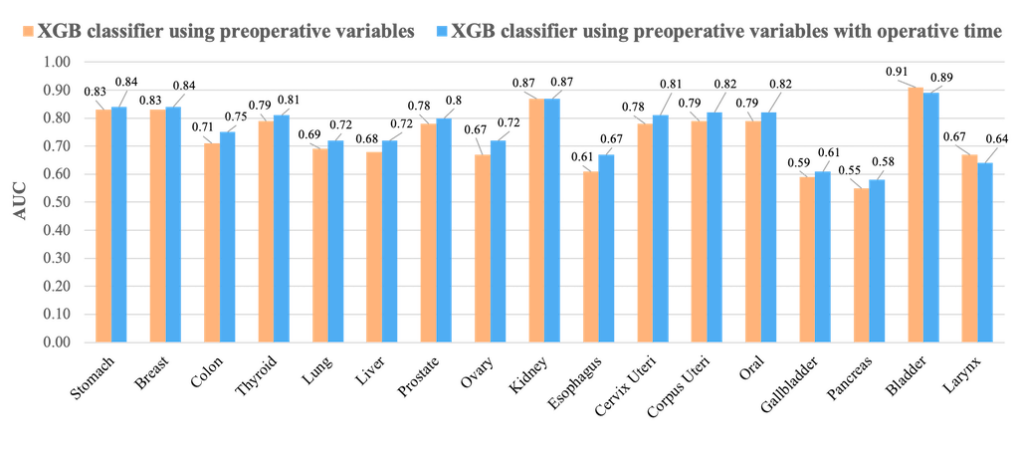
Performance of the extreme gradient boosting (XGB) classifier model for the prediction of prolonged postoperative length of stay by cancer type. The yellow bar indicates the model trained only using preoperative variables and the blue bar represents the model trained with operative time in addition to preoperative variables. AUC: area under the receiver operating characteristic curve.

To evaluate the impact of intraoperative factors on the PPOLOS prediction, we incorporated operative time, a representative indicator of surgery quality, to the models trained on preoperative factors. We evaluated changes in the classification performance of PPOLOS in the model including the operative time. The average AUC increased from 0.74 to 0.76 for all models. Figure 3 shows the prediction performance of XGB classifiers. The yellow bar shows the AUC of the XGB classifier trained with only preoperative variables and the blue bar shows the AUC of the model trained with the operative time in addition to preoperative variables. The model trained with preoperative variables and operative time generally outperformed the models trained without operative time. For bladder and larynx cancer, adding operative time to the models had no benefit in predicting PPOLOS.

## Discussion

### Principal Findings

In cancer surgery, POLOS is both an important indicator for hospital resource use and an indirect predictor of the incidence of surgical complications and recovery of systemic conditions [25,26]. To allocate resources related to cancer surgery properly and predict the time required for recovery after surgery, an evaluation of whether POLOS can be predicted using preoperative data must be performed. Previous statistical studies have focused on identifying risk factors of PPOLOS from among the main variables. However, to develop an application that works on EHRs, an engineering approach is needed. To predict complex outcomes such as PPOLOS, data containing as many variables as possible must be used and data processing must be limited to the application in the medical field.

In the present study, we showed that PPOLOS could be partially predicted using preoperative data from EHRs for various cancer types. Acceptable predictive performance of PPOLOS (AUC >0.8) was observed for stomach, breast, prostate, kidney, and bladder cancers. For lung, liver, ovarian, esophageal, and pancreatic cancers, the predictive performance of PPOLOS was relatively low. During surgeries performed on patients with stomach, breast, prostate, kidney, and bladder cancers, cancer metastasis beyond the affected organ is relatively uncommon and the extent of surgical removal is generally considered to be uniform. In other words, in surgeries for these cancers, organ removal is the most common surgical method, and patients with cancer beyond the organ are often not candidates for surgery as the initial treatment strategy. However, surgeries for lung, liver, ovarian, esophageal, and pancreatic cancers are considered to vary significantly in practice. We assume that the prediction of recovery after surgery and PPOLOS may be possible for cancer surgery with insignificant variations in the surgical methods or with limited extent of the surgical field.

We identified the top-ranking variables associated with PPOLOS for major cancers and confirmed that the following factors correlated with PPOLOS: malnutrition (albumin and globulin), cancer stage, type of surgery, pulmonary function, and BMI [4-8]. Doxofylline and theophylline, which were used for treatment of pulmonary diseases, were associated with PPOLOS after stomach and lung cancer surgeries, respectively. Digestive drugs (famotidine, metoclopramide, and others) and pain medications (acetaminophen and tramadol) also correlated with PPOLOS for various cancers. It could be interpreted that the underlying conditions associated with the use of drugs correlate with PPOLOS, but further research is needed to confirm that the effects of certain drugs contribute to PPOLOS. We further identified that social factors—including marriage, job, and education—affect the hospital discharge time. In a previous study [27], marital status was found to be a factor affecting health care utilization among Medicare beneficiaries.

We categorized the factors that affect PPOLOS and visually identified that there are differences in the relative weight of the factors affecting PPOLOS by cancer types (Figure 2). The effects of surgical factors were relatively high in surgeries for stomach, breast, colon, and kidney cancers. The cancer stage contributed the most to the determination of PPOLOS after thyroid, breast, and ovarian cancer surgeries. For liver cancer, underlying diseases and related laboratory parameters were a major factor when determining PPOLOS. Nutritional factors largely contributed to determining PPOLOS for stomach cancer. Compared with other cancer surgeries, subjective symptoms were an important factor in predicting PPOLOS after breast and thyroid cancer surgeries.

In this study, we aimed to predict the length of the hospital stay after surgery. However, owing to various factors occurring during surgery, it is difficult to determine POLOS. As it is difficult to evaluate the events that occur during surgery using quantitative data from EHRs, we analyzed the effect of operative time. It was observed that the predictive performance of PPOLOS increased markedly for colon, liver, ovarian, and esophageal cancer surgeries. It is believed that a model that predicts POLOS more effectively can be generated by combining preoperative data with intraoperative data, such as vital signs during anesthesia, loss of blood, and surgical instruments used.

Predictive modeling using data from EHRs is expected to improve the quality of health care and allocation of medical resources. However, studies using conventional statistical models have mainly focused on identifying risk factors for length of stay in hospital. Statistical models have limitations in processing numerous unrefined variables and in their application to real-world data. In recent years, machine learning has been used to develop predictive models [11,12]. In this study, XGB classifier and MLP showed slightly better performance than the LR model for surgeries of stomach, breast, colon, thyroid, and lung cancers, which each had more than 4000 cases. Therefore, we believe that machine learning models will be actively used as tools for predicting complex outcomes such as POLOS in the medical field.

One limitation of our study pertains to the fact that variables of data derived from the EHRs of a single cancer center in the Republic of Korea were used. Another limitation is that we used typical methods such as XGB classifier, MLP, and LR. For future study, we need to consider using multicenter EHR data and other methods for analysis. Also, we analyzed data from patients undergoing cancer surgery over a period of 18 years, during which there were likely to have been changes in patient characteristics, clinical practices (such as surgical methods), and patient care after surgery. These temporal trends may have confounded our models’ performance.

If our research results are advanced, we expect to be able to create a model that predicts POLOS before surgery. Following that, it may be possible to build an application into EHRs that can automatically determine the patient’s surgery day by considering the capacity of the ward.

### Conclusions

In our retrospective study, we developed models that predict PPOLOS in patients with cancer and analyzed variables affecting PPOLOS. This approach could help to provide more efficient allocation of medical resources in cancer surgery by embedding machine learning models into the EHR system to support decision making for hospital management.

## Appendix

Multimedia Appendix 1 Characteristics of the cancer population.

Multimedia Appendix 2 Prediction performance of prolonged postoperative length of stay.

Multimedia Appendix 3 Top 10 variables for training the models by cancer type.

## Abbreviations

AUC: area under the receiver operating characteristic curve
EHR: electronic health record
K-CBP: Korea Cancer Big Data Platform
LR: logistic regression
MLP: multilayer perceptron
POLOS: postoperative length of stay
PPOLOS: prolonged postoperative length of stay
ROC: receiver operating characteristic
XGB: extreme gradient boosting
