# Supplementary material for: Prediction of Prolonged Length of Hospital Stay After Cancer Surgery Using Machine Learning on Electronic Health Records: Retrospective Cross-sectional Study
Source: JMIR Med Inform. 2021 Feb 22;9(2):e23147. doi: 10.2196/23147 (PMC7939945; doi:10.2196/23147)
Supplement: Multimedia Appendix 1 [file medinform_v9i2e23147_app1.pdf]

Multimedia Appendix 1: Characteristics of the cancer population

Variable		Total	Stomach	Breast	Colon	Thyroid	Lung	Liver	Prostate	Ovary	Kidney	Esophagus	Cervix Uteri	Corpus Uteri	Oral	Gallbladder	Pancreas	Bladder	Larynx
Subject [N]																			
		42,751	8,929	8,918	7,449	5,071	4,455	1,342	1,054	1,016	767	761	706	535	528	499	365	233	123
**T Stage [N]**																			
	1	16,500	4,715	4,581	894	2,037	1,393	516	20	239	551	269	449	433	150	48	19	157	29
	2	10,419	1,902	3,163	904	93	2,199	504	634	166	58	86	140	44	217	222	25	30	32
	3	11,961	1,571	350	4,119	2,857	583	259	389	607	147	370	5	57	71	207	301	30	38
	4	2,944	741	129	1,435	84	278	59	10	4	11	35	8	0	89	12	15	10	24
	etc	927	0	695	97	0	2	4	1	0	0	1	104	1	1	10	5	6	0
**N Stage [N]**																			
	0	25,922	5,445	5,680	3,559	2,917	2,536	1,261	988	552	740	314	638	465	252	289	0	216	70
	1	10,440	1,638	2,173	2,107	2,144	832	81	63	463	16	370	67	42	94	205	120	9	16
	2	5,003	989	772	1,779	2	921	0	0	0	5	48	1	28	170	5	244	3	36
	3	1,354	853	293	3	0	158	0	0	0	0	29	0	0	12	0	1	4	1
	etc	32	4	0	1	8	8	0	3	1	6	0	0	0	0	0	0	1	0
Female																			
	N (%)	23,835 (55.6)	2,955 (33.1)	8,891 (99.7)	3,032 (40.7)	4,118 (81.0)	1,337 (30.0)	285 (21.2)	0 (0)	1,016 (100)	235 (31.6)	48 (6.3)	706 (100)	535 (100)	146 (27.7)	216 (43.3)	154 (42.2)	58 (25)	11 (8.9)
Age, mean (SD)		56.6 (12.4)	59.1 (12.0)	50.2 (10.5)	60.9 (11.6)	48.1 (11.4)	63.0 (9.5)	57.9 (10.4)	66.6 (7.0)	53.1 (11.4)	55.9 (12.1)	64.4 (8.0)	49.0 (11.4)	54.6 (10.3)	59.8 (13.2)	64.5 (9.1)	64.3 (9.8)	66.2 (10.6)	65.1 (9.3)
LOS [Day], mean (SD)		12.2 (11.3)	12.3 (10.1)	6.8 (6.7)	15.7 (8.7)	4.4 (2.3)	14.8 (11.2)	19.7 (13.9)	11.1 (4.7)	20.1 (13.5)	10.9 (11.4)	25.5 (22.6)	14.5 (12.9)	11.7 (9.1)	24.4 (23.1)	27.9 (17.0)	27.6 (17.0)	8.5 (8.8)	19.7 (17.0)
POLOS [Day], mean (SD)		9.5 (10.2)	10.0 (9.6)	5.4 (6.5)	11.1 (7.8)	3.3 (2.2)	12.0 (10.4)	14.1 (12.3)	9.7 (4.0)	17.0 (12.9)	8.9 (10.8)	22.1 (22.5)	12.2 (12.0)	9.3 (8.0)	22.2 (22.3)	20.7 (14.8)	21.0 (15.1)	7.0 (8.0)	17.6 (16.5)
	Median	7.8	8.0	4.1	8.9	2.9	9.9	10.9	8.0	13.8	7.8	15.8	9.0	7.8	16.9	16.9	16.9	3.9	14.9
**PPOLOS [Day]**																			
	Day threshold	11	10	6	11	4	12	15	9	18	9	24	16	12	27	25	23	11	31
	Ratio [%]	23.7	27.8	26.4	28.8	15.4	26.8	25.3	29.6	26.2	21.1	24.2	21.2	22.4	21.4	25.5	27.1	15.0	19.5
Operative Time [Minute], mean (SD)		180.3 (115.5)	190.3 (71.7)	104.3 (55.4)	205.1 (104.0)	106.7 (53.9)	174.2 (74.3)	267.0 (141.9)	211.7 (72.5)	362.2 (149.7)	194.9 (75.3)	344.8 (108.5)	236.8 (124.8)	219.8 (100.1)	440.2 (307.1)	358.1 (139.2)	369.8 (140.1)	100.5 (125.6)	183.1 (141.9)
