# Supplementary material for: Prediction of Prolonged Length of Hospital Stay After Cancer Surgery Using Machine Learning on Electronic Health Records: Retrospective Cross-sectional Study
Source: JMIR Med Inform. 2021 Feb 22;9(2):e23147. doi: 10.2196/23147 (PMC7939945; doi:10.2196/23147)
Supplement: Multimedia Appendix 2 [file medinform_v9i2e23147_app2.pdf]

Multimedia Appendix 2: Prediction performance of prolonged postoperative length of stay

	XGB[a]				MLP[b]				LR[C]			
	ACC[d]	SPEC[e]	SENS[f]	AUC[g]	ACC	SPEC	SENS	AUC	ACC	SPEC	SENS	AUC
**Stomach**	0.81	0.82	0.47	**0.83**	0.81	0.84	0.53	**0.83**	0.81	0.84	0.53	**0.83**
**Breast**	0.82	0.83	0.45	**0.83**	0.81	0.83	0.45	0.82	0.81	0.84	0.49	0.82
**Colon**	0.74	0.75	0.18	**0.71**	0.75	0.76	0.26	**0.71**	0.73	0.76	0.25	**0.71**
**Thyroid**	0.87	0.88	0.25	**0.79**	0.87	0.89	0.31	**0.79**	0.87	0.89	0.33	0.77
**Lung**	0.72	0.74	0.14	**0.69**	0.72	0.75	0.19	0.67	0.72	0.76	0.25	0.66
**Liver**	0.77	0.8	0.23	0.68	0.74	0.79	0.22	0.67	0.74	0.8	0.29	**0.69**
**Prostate**	0.82	0.82	0.51	0.78	0.77	0.84	0.63	**0.81**	0.81	0.83	0.55	0.78
**Ovary**	0.72	0.75	0.14	**0.67**	0.71	0.80	0.42	**0.67**	0.72	0.79	0.36	0.65
**Kidney**	0.86	0.9	0.54	0.87	0.84	0.93	0.69	0.87	0.88	0.92	0.62	**0.88**
**Oesophagus**	0.74	0.78	0.18	0.61	0.69	0.82	0.48	0.65	0.74	0.8	0.29	**0.66**
**Cervix Uteri**	0.78	0.82	0.29	0.78	0.74	0.88	0.62	0.75	0.8	0.84	0.41	**0.79**
**Corpus Uteri**	0.75	0.79	0.22	**0.79**	0.75	0.83	0.42	0.77	0.78	0.83	0.42	**0.79**
**Oral**	0.82	0.83	0.24	**0.79**	0.72	0.86	0.56	**0.79**	0.79	0.85	0.41	0.75
**Gallbladder**	0.74	0.77	0.11	0.59	0.66	0.80	0.42	0.62	0.72	0.78	0.25	**0.64**
**Pancreas**	0.73	0.77	0.13	0.55	0.61	0.81	0.40	**0.62**	0.68	0.78	0.26	0.56
**Bladder**	0.91	0.93	0.71	0.91	0.87	0.98	0.94	**0.96**	0.88	0.89	0.5	0.93
**Larynx**	0.8	0.83	0.16	0.67	0.70	0.90	0.58	**0.72**	0.82	0.85	0.34	0.70

[a]XGB: extreme gradient boosting classifier, [b]MLP: multiple layer perceptron, [c]LR: logistic regression, [d]ACC: accuracy, [e]SPEC: specificity, [f]SENS: sensitivity, and [g]AUC: area under the receiver operating characteristics curve.
