# Supplementary material for: Prediction of Prolonged Length of Hospital Stay After Cancer Surgery Using Machine Learning on Electronic Health Records: Retrospective Cross-sectional Study
Source: JMIR Med Inform. 2021 Feb 22;9(2):e23147. doi: 10.2196/23147 (PMC7939945; doi:10.2196/23147)
Supplement: Multimedia Appendix 3 [file medinform_v9i2e23147_app3.pdf]

Multimedia Appendix 3: Top 10 variables for training the models by cancer type

Cancer	Stomach			Breast			Colon			Thyroid			Lung		
Model / Rank	**XGB**	MLP	LR	**XGB**	MLP	LR	XGB	MLP	LR	**XGB**	MLP	LR	XGB	MLP	LR
**1**	Globulin	Total gastrectomy	Thoracic & abdominal approach	Urinary symptoms	Urinary symptoms	Breast surgeon: A	Colon Surgeon: B	Colon Surgeon: A	Co-operation with hepato-biliary-pancreatic surgeon	N Stage: 1b	N Stage: 1b	Lobectomy	Albumin	VATS-LLL[a] wedge resection	Age
**2**	Albumin-globulin ratio	Stomach Surgeon: A	Stomach Surgeon: B	Modified radical mastectomy	Breast surgeon: A	Pyridoxine	Co-operation with hepato-biliary-pancreatic surgeon	Laparoscopic Anterior resection	Colon Surgeon: B	Urinary symptoms	Lobectomy	N Stage: 1b	Sex	Age	Corydaline
**3**	Urinary symptoms	Co-operation with another stomach surgeon	Stomach Surgeon: A	Breast surgeon: B	Modified radical mastectomy	Laparoscopic bilateral salpingo-oophorectomy(benign) at same operative episode	Albumin	Co-operation with hepato-biliary-pancreatic surgeon	A-P resection[b] (Miles' operation)	Total thyroidectomy	Globulin	aPTT	Albumin-globulin ratio	Robot assisted thoracoscopic lobectomy and mediastinal lymph node dissection	Bleeding time
**4**	Total gastrectomy	Amylase	Doxofylline	Globulin	Breast surgeon: B	Breast Reconstruction at same operative episode	A-P resection (Miles' operation)	Colon Surgeon: B	Laparoscopic Anterior resection	Albumin-globulin ratio	Thyroid Surgeon A	Radical Operation of Malignant Thyroid Tumor	Globulin	Lung Surgeon A	BMI
**5**	Laparoscopy assisted distal gastrectomy	lidocaine	PMC/Garlic acid	Famotidine	N Stage: 0	Digestive symptoms	Urinary symptoms	A-P resection (Miles' operation)	Co-operation with urological surgeon	Ejection fraction	Occupation	T Stage: 4b	Non-Smoker	BMI	Palpitation
**6**	FEV1[c]	T Stage:4	Albumin	N Stage: 0	Breast surgeon: C	Partial Mastectomy	Laparoscopic Anterior resection	Laparoscopic A-P resection (Miles' operation)	Laparoscopic Total colectomy	N Stage: 0	Urinary symptoms	Co-operation with thoracic surgeon	ANC[e]	Albumin	Megesterol
**7**	ANC	Laparoscopy assisted distal gastrectomy	Acetaminophen	Marriage	Mastectomy (Simple) + expander	BMI[d]	Marriage	Hemicolectomy with lymph node dissection, extended right laparoscopic	Tramadol	Thyroid Surgeon A	Bromelain, dehydrocholic acid, pancreatin, trimebutine, and simethicone	Occupation	Theophylline	Eperisone	Thoracic and abdominal approach
**8**	Albumin	Urinary symptoms	Cilostazol	Breast surgeon: A	Illiteracy	N Stage: 0	N Stage: 0	Abdominoperineal resection of rectum with LN dissection[f]	Albumin	Albumin	N Stage: 0	Thyroid Surgeon A	Planned admission and surgery	Marriage	RBC[g]
**9**	Zolpidem	Thoracic & abdominal approach	Subtotal gastrectomy (Roux-en-Y gastrojejunostomy)	Breast conserving surgery	Breast surgeon: D	Urinary symptoms	Laparoscopic Anterior resection of rectum + LN dissection	Co-operation with another colon surgeon	Hemicolectomy with lymph node dissection, extended right laparoscopic	Drinking	Thyroid Surgeon B	Thyroid Surgeon C	Marriage	Moxifloxacin	Alaxyl granule
**10**	N Stage: 0	Stomach Surgeon: C	Laparoscopic cholecystectomy	Metoclopramide	Pyridoxine	Breast surgeon: E	Urine WBC[h]	T Stage: 4b	T Stage: 4b	Marriage	Pulmonary Function Test	Religion	Hemoglobin	Megesterol	Allopurinol

[a]VATS-LLL: Video-Assisted Thoracic Surgery Left Lower Lobectomy, [b]A-P resection: Abdominoperineal resection, [c]FEV1: Forced Expiratory Volume in one second, [d]BMI: Body mass index, [e]ANC: Absolute Neutrophil Count, [f]LN dissection: Lymph Node dissection, [g]RBC: Red Blood Cell, [h]WBC: White Blood Cells
